# Clinical evaluation of accommodation and ocular surface stability relavant to visual asthenopia with 3D displays

**DOI:** 10.1186/1471-2415-14-29

**Published:** 2014-03-11

**Authors:** Sung wook Wee, Nam Ju Moon

**Affiliations:** 1 Department of Ophthalmology, College of Medicine, Chung-Ang University Hospital, #224-1, Heukseok-Dong, Dongjak-Gu, Seoul 156-755, Republic of Korea

**Keywords:** 3D display, Visual asthenopia, Accommodation, OQAS, Ocular surface stability

## Abstract

**Background:**

To validate the association between accommodation and visual asthenopia by measuring objective accommodative amplitude with the Optical Quality Analysis System (OQAS®, Visiometrics, Terrassa, Spain), and to investigate associations among accommodation, ocular surface instability, and visual asthenopia while viewing 3D displays.

**Methods:**

Fifteen normal adults without any ocular disease or surgical history watched the same 3D and 2D displays for 30 minutes. Accommodative ability, ocular protection index (OPI), and total ocular symptom scores were evaluated before and after viewing the 3D and 2D displays. Accommodative ability was evaluated by the near point of accommodation (NPA) and OQAS to ensure reliability. The OPI was calculated by dividing the tear breakup time (TBUT) by the interblink interval (IBI). The changes in accommodative ability, OPI, and total ocular symptom scores after viewing 3D and 2D displays were evaluated.

**Results:**

Accommodative ability evaluated by NPA and OQAS, OPI, and total ocular symptom scores changed significantly after 3D viewing (p = 0.005, 0.003, 0.006, and 0.003, respectively), but yielded no difference after 2D viewing. The objective measurement by OQAS verified the decrease of accommodative ability while viewing 3D displays. The change of NPA, OPI, and total ocular symptom scores after 3D viewing had a significant correlation (p < 0.05), implying direct associations among these factors.

**Conclusions:**

The decrease of accommodative ability after 3D viewing was validated by both subjective and objective methods in our study. Further, the deterioration of accommodative ability and ocular surface stability may be causative factors of visual asthenopia in individuals viewing 3D displays.

## Background

With the increase of 3D images available for viewing via various multimedia tools, 3D images can be experienced in our daily lives. These developments enable people to watch 3D images for longer periods of time without a break. Recently, there have been various reports of visual discomfort and fatigue regarding the viewing of programs on 3D displays [1]. The discomfort and fatigue are defined specifically as visual asthenopia. There have been a few studies about several ocular factors and symptoms to determine whether visual asthenopia occurs while viewing 3D displays [2-5].

It is widely thought that the conflict between accommodation and vergence is a possible factor influencing visual fatigue. Several studies have examined the accommodation-vergence conflict and its impact on visual fatigue with viewing 3D displays [2,3]. Also, the present authors reported that the amplitude of accommodation and convergence decreased and correlated significantly to increased visual asthenopia after 3D viewing in the previous publication [4]. We identified that accommodation and vergence conflict may occur within the subjects viewing programs on 3D displays. This may be due to an increase in near visual tasks which in turn, may lead to eventual deterioration of the capability to accommodate and converge, contributing to the development of visual asthenopia.

Clinically, accommodative and convergence amplitudes are usually measured using a subjective push-up test. However, a subjective push-up method does not provide sufficient reproducibility because of the subjectivity of the examiner and examinees [6]. Therefore, an objective measurement is required for reliable examination. The Optical Quality Analysis System (OQAS®, Visiometrics, Terrassa, Spain) is the instrument used for objective measurement of optical quality [7], intraocular scattering [8] in a clinical setting. In addition to optical quality measurement, this system also provides an objective estimation of accommodation.

In the present study, we used the OQAS to measure objective accommodation amplitudes before and after viewing displays and to verify our previous results on the decrease of accommodative capability that is believed to develop visual asthenopia after viewing 3D displays. We excluded the vergence factor in this study because there have been few studies that objectively measured vergence amplitude to our knowledge.

Besides the conflict between accommodation and vergence, the ocular surface instability may be a potential source of visual asthenopia. In the study of visual display terminal (VDT) syndrome, dry eye was found to be a potential primary cause of ocular fatigue. The mechanism underlying dry eye causing ocular fatigue, such as that experienced when using video displays, is a decreased blink rate and an increased exposure of the ocular surface, causing desiccation of the eye [9]. We utilized the ocular protection index (OPI) to measure ocular surface stability in this study. The OPI was developed to quantify the interaction between blinking and the tear film [10]. It may therefore provide a framework to assess the effects of tear film instability while viewing displays, and whether the visual asthenopia with 3D viewing is associated with ocular surface instability.

In this study, we performed a further evaluation on 3D displays based on a previous publication [4] to confirm the association between accommodation and ocular asthenopia by measuring objective accommodative amplitude with OQAS. We also investigated correlations among the accommodation, the ocular surface instability and visual asthenopia while viewing 3D displays.

## Methods

### Design

Prospective case study in university hospital.

### Subjects

Fifteen young healthy volunteers signed informed consent forms after being provided with a detailed explanation of the study. The study protocol was in accordance with the Declaration of Helsinki and was approved by the institutional review board of Chung-Ang University Hospital, Seoul, Korea. There was no other ocular disease or surgical history in any of the volunteers included in the study. All cases with disorders of accommodation, vergence, and stereoacuity were excluded.

The subjects watched programs on the 3D and 2D displays with the same content using a double-blind method in a random order. We used a commercial Blu-ray disc IMAX® Space Station (Warner Bros., California, USA) as a display for both the 3D and 2D versions of the experiment. A 23 inch-sized 3D display-compatible self-emitting plasma display panel (3D Cinema monitor**®** 23MD53D**,** LG electronics, Seoul, Korea) with the following specifications: aspect ratio: 16:9, spatial ratio: 1920 × 1080 and environmental luminance at the screen: 250 lux was used. The subjects watched these 3D displays for 30 minutes with film patterned retarder glasses (FPG-2000®, LG electronics, Seoul, Korea). The distance between the eyes and monitor was 70–90 cm, and the exact distance was determined according to the subject’s preference. Also, the subjects watched the same display in 2D for 30 minutes with placebo glasses as controls.

### Main outcome measures

Several ocular factors were evaluated 3 times; before, after 3D viewing, and after 2D viewing. Each examination was separately performed with a one-week interval.

Accommodative ability was evaluated by two methods - the subjective near point of accommodation and OQAS - to ensure reliability. The monocular near point of accommodation (NPA) was obtained using Donder’s subjective push-up method. A 20/30 single letter on a fixation stick, approximately 50cm from the subject, served as the target, and was moved gradually closer to the subject at about 5.0 cm/sec, until the subject noticed the target starting to blur. This was considered the endpoint. The tests were performed with the subject’s distance correction.

Another measurement of accommodation was obtained by an OQAS. With full correction of distant visual acuity, subjects were seated at the instrument with their head stabilized in the instrument’s chin rest and forehead strap. After dimming the lights in the room, subjects viewed the 20/40 sized near target at 33cm. Accommodation was stimulated by means of the push up method in the range from 0 to 5 D with a 1-D step. During the near target moved toward the subject and triggered accommodative stimulus, the subject was asked to focus on the target and keep it clear while the objective refraction measurement was made. This measurement was considered the amplitude of accommodation in diopters.

Tear break up time (TBUT) was measured with a Fluorescein® strip (Haag-Streit International, Köniz-Bern, Switzerland) coated with one drop of balanced salt solution (BSS®, Alcon laboratories, Fort Worth, Texas, USA). After applying the strip to the inferior conjunctival fornix, subjects were instructed to maintain their normal blinking frequency for several seconds. After the fluorescein solution spread equally onto the corneal surface, subjects were required to keep the eye open until the first defect in the tear film occurred. The moment that the first defect of tear film occurred was considered the TBUT. The slit lamp examination was done at 10x magnification [11].

The interblink interval (IBI) was measured by calculating the average blink rate for five minutes. The total blink count was calculated using a video camera (SMX-F70®, Samsung Electronics, Korea) recording to ensure accuracy. The OPI was calculated by dividing the TBUT by the IBI (OPI = TBUT/IBI) [12]. Unlike the measurement of other factors, the IBI results after viewing displays were actually measured during the last five minutes of the experiment because the IBI value is closely related to concentration on the displays.

All measurements were repeated three times for each eye tested except the IBI, and results were reported as the mean value. All measurements that required a single eye examination were performed on the right eye only. All subjects were examined by a single examiner after the full correction of refractive error with glasses.

Subjective ocular discomfort was evaluated with the questionnaire proposed by Sheedy et al [12]. We added dizziness as another item to the established questionnaire because the subjects complained of dizziness while viewing the programs on the 3D displays in the preliminary study. The questionnaire consisted of the ten symptoms. The symptom sensation questionnaire contained six identical analog scales (0 = none and 6 = too severe to stand) on which the subject recorded the extent of each of the symptoms. The questionnaire was administered before and after viewing displays, and total sum of the scores of ten symptoms was calculated.

### Statistical analysis

Statistical analyses were performed using statistical software (SPSS for Windows, V.16.0 SPSS Science, Chicago, Illinois, USA). For all tests, the significance level was set at *p* < 0.05. Changes in the accommodative ability, OPI, and the total score of ocular symptoms before and after viewing displays were compared using the Wilcoxon signed-rank test. Correlations among accommodative ability, OPI, and ocular symptoms were analyzed using the Spearman’s correlation test.

## Results

Ten males and five females with a mean age of 25.6 ± 2.10 years (aged 23 to 30 years) were enrolled. We compared the data after 3D and 2D viewing with single baseline data that was measured at least 1 week before watching either of the displays.

Table 1 summarizes the results for the several ocular factors and symptoms before and after 3D and 2D viewing. The NPA, the accommodative power evaluated by OQAS, IBI, OPI, and the total ocular symptom scores changed significantly after 3D viewing compared to baseline data (p = 0.005, 0.003, 0.001, 0.006, and 0.003, respectively), while no significant change was observed after 2D viewing. TBUT yielded no significant change after 3D viewing and 2D viewing compared to baseline data. In the comparison between the data of post 3D viewing and post 2D viewing, the NPA, OQAS, IBI, OPI, and the total ocular symptom score changed significantly after 3D viewing compared to 2D viewing (p = 0.004, 0.037, 0.005, 0.017 and 0.004, respectively).

**Table 1:** Results of ocular factors before and after 3D and 2D viewing

**Factors**	**Baseline**	**Post-3D**	P value*	**Post-2D**	**P value**^**†**^	**P value**^**‡**^
NPA^§^(cm)	10.75 ± 1.86	11.52 ± 1.53	0.005	9.95 ± 2.51	0.167	0.004
OQAS^||^ (diopters)	2.35 ± 0.38	2.0 ± 0.46	0.003	2.32 ± 0.47	0.666	0.037
TBUT^#^(sec)	7.33 ± 1.68	7.60 ± 1.12	0.604	7.67 ± 1.54	0.672	0.923
IBI^¶^(sec)	3.84 ± 0.90	5.54 ± 0.87	0.001	4.52 ± 0.54	0.027	0.005
OPI^††^	2.0 ± 0.61	1.39 ± 0.22	0.006	1.72 ± 0.40	0.140	0.017
SUM^**^	0.80 ± 0.94	5.67 ± 5.45	0.003	2.07 ± 2.89	0.196	0.004

NPA^§^, near point of accommodation; OQAS^||^, accommodative power calculated using the optical quality analysis system; TBUT^#^, tear break-up time; IBI^¶^, interblink interval; OPI^††^, ocular protection index; SUM^**^, total ocular symptom score.

P value*, Comparison between ophthalmologic factors of post-3D and Baseline.

P value^†^, Comparison between ophthalmologic factors of post-2D and Baseline.

P value^‡^, Comparison between ophthalmologic factors of post-3D and post-2D.

In the comparative analysis of ocular symptoms, the symptoms of pain, dullness, irritation, dryness, blurred vision, and total ocular symptom scores were significantly aggravated after 3D viewing compared to baseline data. In the analysis between ‘after 3D viewing’ and the ‘after 2D viewing’, the symptoms of pain, dullness, dryness, tearing, and total ocular symptom scores were significantly aggravated. Table 2 summarizes the results for the subjective ocular symptoms before and after 3D viewings, and after 2D viewings.

**Table 2:** Comparison between parameters representing subjective ocular symptoms before and after watching 3D and 2D displays

**Symptoms**	**Baseline**	**Post-3D**	P value*	**Post-2D**	**P value**^**†**^	**P value**^**‡**^
Pain	0	0.67 ± 1.07	0.041	0	1.0	0.041
Dullness	0.20 ± 0.40	0.87 ± 1.09	0.041	0.27 ± 0.57	0.655	0.024
Headache	0.07 ± 0.25	0.87 ± 1.09	0.072	0.60 ± 1.08	0.102	0.102
Diplopia	0	0.13 ± 0.34	0.157	0.20 ± 0.54	0.180	0.317
Burning	0	0.33 ± 0.60	0.059	0.07 ± 0.25	0.317	0.102
Irritation	0.27 ± 0.44	0	0.046	0	0.046	1
Dryness	0.20 ± 0.40	1.20 ± 1.11	0.011	0.53 ± 0.62	0.059	0.040
Tearing	0.07 ± 0.25	0.4 ± 0.61	0.096	0	0.317	0.034
Blurred vision	0	0.87 ± 1.09	0.017	0.33 ± 0.79	0.102	0.071
Dizziness	0	0.33 ± 0.79	0.102	0.07 ± 0.25	0.317	0.102
SUM^§^	0.80 ± 0.94	5.67 ± 5.45	0.003	2.07 ± 2.89	0.196	0.004

SUM^§^, total ocular symptom score.

P value*, Comparison between subjective symptoms of post-3D and Baseline.

P value^†^, Comparison between subjective symptoms of post-2D and Baseline.

P value^‡^, Comparison between subjective symptoms of post-3D and post-2D.

**Figure 1:**
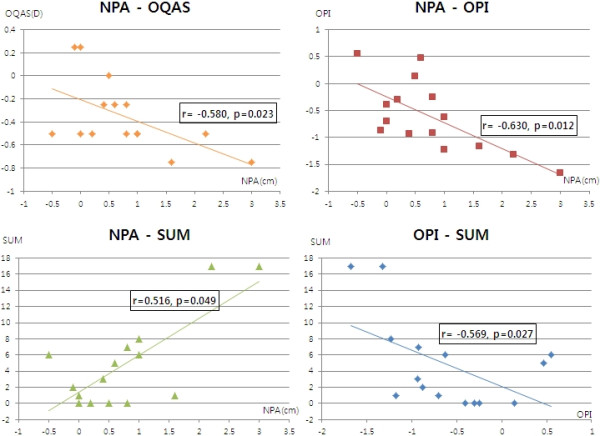
**Correlation analyses of the changes of several ocular factors and symptoms with 3D viewing.** (Top left and right) The change of the near point of accommodation (NPA) negatively correlated with the change of accommodative power evaluated by OQAS (OQAS) (r = -0.580, *p* = 0.023) and ocular protection index (OPI) (r = -0.630, *p* = 0.012). (Bottom left) The change of NPA positively correlated with the total ocular symptom scores (SUM) (r = 0.516, *p* = 0.049). (Bottom right) The change of OPI negatively correlated with SUM (r = -0.569, p = 0.027) with 3D viewing.

In the correlation analysis with 3D viewing, the NPA change negatively correlated with the change in accommodative power evaluated by OQAS (r = -0.580, *p* = 0.023) and OPI (r = -0.630, *p* = 0.012). In addition, the NPA change positively correlated with the total ocular symptom scores (r = 0.516, *p* = 0.049). The change in OPI negatively correlated with the total ocular symptom scores (r = -0.569, p = 0.027) with 3D viewing (Figure 1).

## Discussion

There have been several studies about the mechanism underlying visual asthenopia development when viewing 3D displays. Discrepancy between accommodative and vergence stimuli is common in stereoscopic images, because accommodation should respond to the screen/image position but disparity of the two images for both eyes, vergence stimulus, varies over time [13-15]. This discrepancy can result in visual stress and fatigue. On the contrary, there have been studies insisting that there is little discrepancy between accommodation and convergence during the viewing of 3D images and the motion sickness induced by 3D video clips might be caused by the sensory conflict as a disagreement between visual and vestibular inputs [16,17]. We think that this dispute is unlikely to be settled for a long time to come and more studies and prolonged discussion should be followed.

In the previous study, the authors identified that there is a decrease of accommodation and convergence, due to cumulative fatigue with discrepancy between accommodative and vergence stimuli from 3D displays [4]. In this study, we utilized OQAS to measure objective accommodation amplitudes to make up for the weakness within the subjective push-up method. In the present study, the results of the accommodative ability measured by evaluating the NPA and OQAS were significantly correlated with 3D viewing. This implies that the finding of previous study that the decrease of accommodative ability with 3D viewing was verified by objective measurement.

It is important to assess the repeatability of the measurements with the OQAS to confirm the applicability of the data. In previous studies, it has been demonstrated that the device has reliable repeatability [18-20]. Kamiya et al. [18] reported several parameters representing optical quality measured by OQAS showed a narrow 95% limit of agreement, demonstrating good repeatability of optical quality measurements. Also, Vilaseca et al. [19] reported that acceptable intra- and intersession repeatability was observed and the realignment of the eye did not introduce any variability in the measurements with OQAS.

Ocular surface instability may be considered another causative factor of 3D visual asthenopia. If one had intensive near visual work continuously with any displays, the decrease in the blink rate and enhanced destruction of the tear film may result in ocular surface instability [21,22]. We hypothesized that increased concentration on the near visual stimuli of 3D displays may decrease the blink rate more than the stimuli on 2D displays, resulting in aggravation of dry eye symptoms. In the present study, we utilized the OPI to quantify the interaction between blinking and the tear film. Lower OPI scores may reflect deteriorated ocular surface stability. While viewing 3D displays, the OPI score decreased significantly followed by a significant increase in the IBI, whereas TBUT did not change significantly. This implies that a decreased blink rate rather than a stable TBUT while 3D viewing may result in a decreased OPI score. In addition, the accommodative ability measured by NPA and the OPI score decreased with significant correlations. We therefore thought that increased concentration on accommodative stimulus with 3D displays may result in a decreased blink rate and OPI score, ultimately leading to ocular surface instability. Further studies might be needed to validate this relation because it is difficult for the authors to figure out the underlying mechanism associated with accommodation and blinking rate with 3D displays in this study. Although it is unclear that there is direct association between accommodation and blink rate, the present study will be valued because this is the first study to discuss the ocular surface factors that may trigger visual asthenopia with 3D viewing.

In the comparative analysis of ocular symptoms, pain, dullness, irritation, dryness, blurred vision, and total ocular symptom scores were significantly aggravated after 3D viewing compared to baseline data. Significant differences were also noted within above-mentioned symptoms except irritation and blurred vision for the ‘after 3D viewing’ and the ‘after 2D viewing’ analyses. Although it is not clear why these significant changes occurred in the limited specific symptoms, we may certainly infer that visual asthenopia may be developed with 3D viewing, as compared with 2D viewing.

In the correlation analysis of the change of ocular factors and symptoms, a decrease in the accommodative ability and the OPI score, and an increase in the total ocular symptom scores showed significant correlations with 3D viewing. This implies that the deterioration of accommodative ability and ocular surface stability may play an important role in the development of visual asthenopia while viewing 3D displays.

We may propose the possibility of developing 3D technology with less discomfort from the currently confirmed results of our studies. Further study regarding reduction of excessive accommodation and concentration on near stimulus of 3D display should be carried out. For example, the 3D images with uncrossed disparity that are perceived to be located behind the screen may trigger fusional divergence rather than fusional convergence. As convergence, accommodation and miosis occurs simultaneously as a near vision complex, the images with uncrossed disparity may not cause unnecessary accommodation and may reduce ocular asthenopia [23]. Furthermore, to investigate more detailed aspects regarding the incidence of asthenopia due to 3D displays, subjects with abnormal accommodative ability and ocular surface instability such as presbyopia and dry eye syndrome should be included in future studies.

The small number of participants and lack of subjects with problems of accommodation and ocular surface stability may be limitations. Also, as the present study did not include the elderly and children, further study with groups with a broader range of age may result in different findings. In addition, unlike the measurement of NPA, the objective accommodative ability measured by OQAS did not show a significant correlation with the total ocular symptom score in the analysis. These factors should be considered as limitations of our study.

## Conclusions

The accommodative ability evaluated by measuring NPA and OQAS and ocular surface stability evaluated by measuring the OPI decreased significantly after 3D viewing as compared to 2D viewing. The total score of ocular symptoms increased significantly in subjects when viewing 3D displays compared to 2D displays. Therefore, decreased accommodative ability and ocular surface stability may influence the development of visual asthenopia when viewing 3D displays.

## Abbreviations

OQAS: Optical Quality Analysis System; VDT: Visual display terminal; OPI: Ocular protection index; NPA: Near point of accommodation; TBUT: Tear break up time; IBI: Interblink interval; SUM: Total ocular symptom scores.

## Competing interests

There are no competing interests for any author in the present study.

## Authors’ contributions

SWW: Co-author. 1) Substantial contributions to conception and design, or acquisition of data, or analysis and interpretation of data. 2) Substantial contributions to Drafting the article or revising it critically for important intellectual content. 3) Final approval of the version to be published. NJM: Corresponding author. 1) Substantial contributions to conception, design, and interpretation of data. 2) Revising the article critically for important intellectual content. 3) Final approval of the version to be published. Both authors read and approved the final manuscript.

## Pre-publication history

The pre-publication history for this paper can be accessed here:

http://www.biomedcentral.com/1471-2415/14/29/prepub
